# Cardiac arrhythmias and genetics – current stage

**DOI:** 10.1515/medgen-2025-2006

**Published:** 2025-04-08

**Authors:** Sven Dittmann, Janis Kerkering, Schulze-Bahr

**Affiliations:** Institute for Genetics of Heart Diseases (IfGH) University Hospital Münster (UKM) Albert-Schweitzer-Campus 1 (Gebäude D3) 48149 Münster Germany; Institute for Genetics of Heart Diseases (IfGH) University Hospital Münster (UKM) Albert-Schweitzer-Campus 1 (Gebäude D3) 48149 Münster Germany; Institute for Genetics of Heart Diseases (IfGH) University Hospital Münster (UKM) Albert-Schweitzer-Campus 1 (Gebäude D3) 48149 Münster Germany

**Keywords:** Inherited cardiac arrhythmias, Cardiac ion channel disorders, Cardiogenetics, Long-QT syndrome, Sudden cardiac death, SCD

## Abstract

Recently, cardiogenetics is a rapidly developing medical section combining cardiovascular and genetic knowledge. Inherited forms of cardiac arrhythmias are typically rare diseases (prevalence < 1:2,000) and may occur in a sporadic or familial manner, here mostly in an autosomal dominant form. They are also called “primary electrical heart disorders” due to the ECG-based diagnosis and mainly normal cardiac imaging, i.e. absence of structural heart abnormalities. Their genetic basis is heterogeneous, still incomplete (variant detection rates between 10 % and 80 %) and mostly related to cardiac ion channel genes and related regulatory units. So far, the utility of polygenic risk scores is under current evaluation. Clinical disease expressivity may range from non-penetrance to high penetrance, indicating the importance of additional clinical modifiers (genetic and non-genetic) that modulate phenotypic signs. Occurrence of symptoms, as typical for other ion channel disorders (e.g., epilepsy), also depends on exposure to specific and often genotype-related environmental triggers, that enhance the occurrence of clinically relevant and potentially life-threatening arrhythmias.

In the following, the main focus is on cardiac ion channel disorders, with regard to some general genetic aspects and current guidelines indicating the value of genotyping to support early disease recognition, confirmation of diagnosis and prevention of severe cardiac events.

## 1. Introduction

The familial occurrence of several cardiac arrhythmias has been recognized since >60 years and has led to the first identification of cardiac ion channel genes in the mid-1990s of the last century. Most importantly, this gave important pathophysiologic insights into the arrhythmogenesis by in-depth understanding of structure-function relationships of cardiac ion channel components have been identified that are determinants of cardiac action potential generation and inherited forms of primary electrical heart diseases. Recently, patient-derived disease models using differentiated cardiomyocyte-like cells from human-induced pluripotent stem cells (hiPSCs) have provided tremendous insights into myocellular electrical dysfunction and therapeutic pathways [1, 2].

Apart from a few founder population variants, the majority of identified pathogenic variants are “private” (i.e., family specific). In addition, nearly every inherited arrhythmia is genetically heterogeneous and often related to 5–10 ion channel genes. For some of them, ion channel dysfunction may result in different phenotypes (e.g., the sodium channel gene *SCN5A*: a loss-of-function due to a pathogenic gene variant results in Brugada syndrome, cardiac conduction disease or dilatated cardiomyopathy, whereas a gain-of-function variant results clinically in a long-QT syndrome phenotype) or even in overlapping ones. The significant allelic heterogeneity and variety of ACMG class 4/5 variants (e.g., for long-QT syndrome: >650 different KCNQ1 (LQT1 subtype) variants, >300 different KCNH2 (LQT2) variants) with an overall high variant detection rate (70–80 %; Table 1). In contrast, in some primary electrical disorders the variant detection rate (“sensitivity of a genetic test”) is still low (e.g., atrial or ventricular fibrillation, 10–20 %) raising the issue of clinical phenotypic or mimicking conditions. Therefore, precise knowledge and clinical recognition of the genetic forms together with an evaluated family history are essential, including differentiation from non-genetic forms.

In the light of existing genetic heterogeneity, but also of unforeseen genetic complexity in known disease genes, next-generation sequencing (NGS) technologies have improved modern genetic diagnostics in terms of accuracy, comprehensivity, analysis duration and costs. Together with sufficient bioinformatic variant assessment and pathogenicity prediction, the parallelized gene analysis (e.g., several hundreds of genes for a distinct phenotype in a single analysis run) has replaced previous DNA analysis by Sanger sequencing, which is currently used for secondary validation of NGS findings or family variant detection. Very recently, in addition to targeted gene panels or whole exome sequencing (WES), whole genome sequencing (WGS) has been found to be superior to WES by the detection of potentially structural variants (SVs, CNVs) and variants in relevant, but non-coding gene regions [3, 4].

**Table 1:** Inherited forms of cardiac arrhythmias and potential implications of genetic testing

Disease	Variant Detection Rate (Sensitivity)	Implication of a pathogenic genetic finding		Reference
		Diagnostic	Therapeutic/ Prognostic	
Long-QT syndrome (LQTS)	70–80 %	+++	+++ / +++	ClinGen MonDO: 0002442
Brugada syndrome (BrS)	20–30 %	+	+ / ++	ClinGen MonDO: 0015263
Catecholaminergic Polymorphic Ventricular Tachycardia (CPVT)	50–60 %	+++	++ / +	ClinGen MonDO: 0017990
Short-QT syndrome (SQTS)	25 %	+	+ / +	ClinGen MonDO: 0000453
Idiopathic ventricular fibrillation (IVF, SCA)	20–30 %	++	++	
Early repolarization syndrome (ERS)	?	-	- / -	
Short-coupled Torsade de pointes tachycardia (scTdP)	?	+	+ / -	
Sinus node disease (SND)	?	-	+ / -	
Cardiac conduction disease (CCD/PCCD)	10–20 %	+	+ / +	
Atrial fibrillation	?	-	- / -	
Sudden cardiac death, unclear (SIDS, SADS, SUDS, SUNDS, SCD)	10–30 %	++		

CCD, “cardiac conduction disease”; PCCD, “progressive cardiac conduction disease”; SND, “sinus node disease”; SADS, “sudden arrhythmic death syndrome”; SCA, “sudden cardiac arrest”; SCD, “sudden cardiac death”; SIDS, “sudden infant death syndrome”; SUDS, “sudden unexpected death syndrome”; SUNDS, “sudden unexpected nocturnal death syndrome”.

However, apart from delineating the genomic complexity of monogenic cardiac disorders, it is likely that upon NGS analyses (independent of the number of genes being bioinformatically analyzed), cardiovascular genes not being previously linked to the patient’s phenotype will be addressed and novel genes (of uncertain, but potentially disease-related significance) will be identified. Importantly, the diagnostic set-up will be left, and additional confirmatory experimental steps are required to establish the potential disease relationship before the genetic results will be communicated to the patient and their physicians.

The following guidelines and recommendations are useful for further reading:

Genetic Testing for Heritable Cardiovascular Diseases in Pediatric Patients. A Scientific Statement From the American Heart Association [5],
2020 APHRS/HRS expert consensus statement on the investigation of decedents with sudden unexplained death and patients with sudden cardiac arrest, and of their families [6],
German consensus document “Postmortale molekulargenetische Untersuchungen (Molekulare Autopsie) bei kardiovaskulären und bei ungeklärten Todesfällen, Konsensuspapier der Deutschen Gesellschaft für Kardiologie (DGK), Deutschen Gesellschaft für Pädiatrische Kardiologie (DGPK), Deutschen Gesellschaft für Humangenetik (GfH), Deutschen Gesellschaft für Pathologie (DGP), Deutschen Gesellschaft für Rechtsmedizin (DGRM)” [7],
European Heart Rhythm Association (EHRA)/Heart Rhythm Society (HRS)/Asia Pacific Heart Rhythm Society (APHRS)/Latin American Heart Rhythm Society (LAHRS) Expert Consensus Statement on the state of genetic testing for cardiac diseases [8],
2022 ESC Guidelines for the management of patients with ventricular arrhythmias and the prevention of sudden cardiac death [9],
German consensus document “Gendiagnostik bei kardiovaskulären Erkrankungen. Konsensuspapier der Deutschen Gesellschaft für Kardiologie (DGK), der Gesellschaft für Humangenetik (GfH) und der Deutschen Gesellschaft für Pädiatrische Kardiologie (DGPK)” [10].

**Table 2:** Current recommendations for genetic testing of a proband with an inherited cardiac arrhythmia [8, 10]

Recommendation Class for Genetic Testing	Inherited Arrhythmia Form	Genetic Result is part of a Diagnostic Score
I	Long-QT syndrome (LQTS) Brugada syndrome (BrS) with type 1-ECG Catecholaminergic Polymorphic Ventricular Tachycardia (CPVT) Short-QT syndrome (SQTS) Familial/syndromic (progressive) cardiac conduction disease (PCCD/CCD) Idiopathic ventricular fibrillation (IVF, SCA), early onset	X X X X
IIa	Idiopathic ventricular fibrillation (IVF, SCA), adult onset Short-coupled torsade de pointes tachycardia (scTdP) MEPPC (Multiple ectopic Purkinje-related premature ventricular contractions)	
IIb	Brugada syndrome (BrS) with type 2/3-ECG Sudden cardiac death, unclear (SIDS, SADS, SUDS, SUNDS, SCD) Familial sinus node disease (SND) Familial atrial fibrillation (AFIB)	X
III	Early repolarization syndrome (ERS)	

CCD, “cardiac conduction disease”; PCCD, “progressive cardiac conduction disease”; SND, “sinus node disease”; SADS, “sudden arrhythmic death syndrome”; SCA, “sudden cardiac arrest”; SCD, “sudden cardiac death”; SIDS, “sudden infant death syndrome”; SUDS, “sudden unexpected death syndrome”; SUNDS, “sudden unexpected nocturnal death syndrome”;

In these references, the strength of evidence for genetic testing and its recommendation concerning specific inherited arrhythmia types can be at least categorized in: class I recommendation (i.e., “recommended to do”), class IIa (“should be considered to do”), class IIb (“may be considered to do”), and class III (“not recommended to perform”).

In Table 2, the current recommendations for specific inherited arrhythmias are summarized.

## 2. Cardiogenetic services – an upcoming need

The first descriptions of familial arrhythmia and cardiomyopathy forms and the thereby observational evidence for an inherited basis were followed by the first discoveries and descriptions of the underlying cardiovascular genes and causative variants and, in the recent light of rapidly evolving and developing sequencing techniques, resulted in a dramatically increasing understanding of cardiovascular genetics today. In consequence, due to significantly reduced analysis costs and test durations and due to the increased availability, cardiogenetic testing is becoming more and more reality in clinical routine practice. In Germany, these processes are slowly developing and suffer from structural and regulatory limitations. So far, the number of specialized and thereby interdisciplinary cardiogenetic centres is mainly limited at leading academic institutions. The aim of such centres is a directed and better focussed diagnosis of single-gene cardiovascular diseases that are often unrecognized and initiation of a subsequent genetic testing, adequate disease risk assessment and therapy upon clinical features and, finally, familial cascade screening for preventive disease detection. Hereby, the adjusted interpretation and application of genetic information for the prevention, diagnosis, and treatment of cardiovascular disease will become an important part in cardiovascular medicine with the potential of a high impact on patient and family care.

In consequence, cardiovascular genetics is a rapidly evolving specialty of cardiovascular medicine that has not historically been addressed in traditional cardiology, pediatric cardiology or genetic fellowship trainings; recently, several attempts have been performed to fill these gaps (e.g., EHRA Educational courses and EP fellowship or webinars by the ERN Reference Network Guard Heart).

In line with the majority of monogenic disorders, inherited cardiovascular conditions are also genetically heterogeneous and pathogenic variants are often ‘private’, i.e. family-specific. Also, incomplete and age-specific penetrance are common, in particular in inherited cardiomyopathies forms, but also with regard to disease presentation in inherited arrhythmia forms. Therefore, clinical counselling and therapeutic recommendations mainly depend on the individual clinical expressivity of a pathogenic genetic variant, with respect to genetic subtype. Of note, some of the current diagnostic scores for inherited arrhythmias already implemented a positive genetic result (Table 2), often by scoring the presence of a pathogenic variant being equivalent to the full clinical diagnosis. E.g., in the presence of incomplete disease penetrance (i.e., absence of clinical signs) the identified pathogenic gene variant is quite obvious to support for a clinical diagnosis rather than to make the diagnosis itself.

In **interdisciplinary cardiogenetic centres**, experienced cardiologists (cardiac electrophysiology, cardiac imaging), pediatric cardiologists and genetic counsellors are under one roof to perform a high and multivariate patient care, that is complemented by an accredited genetic laboratory. In the EU, labs and tests should be accredited in their jurisdiction; the majority of offered genetic tests, so far, have been developed developed in-house (so-called laboratory-developed tests or LDTs) and are validated as well as inspected by certified national organizations.

Within the available **types of cardiovascular genetic testing**, single-gene tests (by Sanger sequencing) have been replaced by multi-gene panels (MGPS) using next-generation sequencing (NGS) with a selection of several hundred of genes and might be useful, in particular, if there is a diagnostic uncertainty that can be often found in the clinical overlap between arrhythmogenic cardiomyopathy forms and ventricular arrhythmias. Here, a broader testing strategy with a larger gene panel may be of higher utility. Larger panels or whole exomes (WES) are not likely to increase the variant detection rate in case of a confirmed cardiovascular diagnoses but may increase the amounts of detected variants of unknown clinical significance (ACMG class 3 variants) and secondary or incidental findings that each require specific, professional frameworks of phenotype assessment, counselling and finally patient advice. With regard to potentially causative cardiovascular genetic variants, cardiogenetic centres and their accredited labs are likely to be experienced in gene-specific single nucleotide- (SNVs) and copy number variants (CNVs) detection; since whole genome sequencing (WGS) is not routinely applied in adult cardiovascular clinical care, but can detect a wider array of types of genetic abnormalities (small CNVs, repeat expansions, regulatory variants). Targeted panels with a dedicated CNV analysis are currently being routine, also with regard to lower lab costs, good variant detection rate (sensitivity) and a preformed amount of bioinformatic sequence interpretation.

Typical issues to perform **molecular genetic testing** in the families’ index patient (proband) are (1) *to unravel the cause of the cardiovascular disease and symptoms,* (2) *to provide a diagnosis,* (3) *to provide prognostic information and adjust clinical management*, and (4) *to identify disease-causing triggers, in particular in inherited arrhythmia syndromes.* Significant variability in genetic testing access and provisions between countries is mainly due to the availability of dedicated cardiogenetic laboratories and due to reimbursement issues [11]; e.g., in Germany, there are currently significant differences between private and non-private health care insurances.

With regard to their heritability, i.e. the fraction of **phenotypic variation** caused by genetic variation, for nearly all inherited arrhythmia syndromes genetic heterogeneity exists and, even when applying a NGS approach, the variant detection rates are between 10 % to 80 %, either due to incomplete genetic knowledge of causative genes or due to phenocopies that might not have been considered before genetic testing. With regard to phenotypic variation of an inherited arrhythmia syndrome, twin studies and genome-wide association studies (GWAS) showed that many, specific ECG parameter and traits have a substantial heritability and might explain differing disease expressivity [12–14], but, since often the mechanism underlying this heritability has not been unraveled and the phenotypic effect appeared too small, routine genotyping of SNP variation in the context of a genetic/polygenic risk score (GRS/PRS) is not performed. In this line, recent cohort studies from the UK Biobank [15, 16] with >26,000 participants (with whole-genome sequencing and ECG data) identified 160 carriers of putative pathogenic rare variants in 10 LQTS genes and individuals with a QTc interval >480 ms, 23.7 % were carriers of a monogenic, rare variant or had a PRS (21 % top decile); the PRS resulted from 54 independent genomic loci (33 known and 21 novel) reported to be associated with the QTc interval and resulted in a SNP heritability of 0.24 [15]. Variant interpretation in the clinical setting is greatly enhanced by the use of disease-specific, multidisciplinary teams that could include clinical disease experts, clinical geneticists, genetic counsellors, and molecular geneticists.

Another important issue for interdisciplinary cardiogenetic centres might be the **reinterpretation and reclassification of identified genetic variants** in the light of specific phenotypic information; a recent study [17] thereby showed a reclassification rate of >36 % (among them: cardiomyopathies variants with a rate of 28 %, cardiac channelopathies with 38 %), comparable to other studies in survivors of cardiac arrest (38 %), pediatric epilepsy (36 %) or dilated cardiomyopathy (30 %). Reclassification of variants was related to additional information about other affected cases and variant co-segregation, experimental loss-of-function evidence and other functional data upon current ACMG criteria mainly leading to a downgrading of pathogenicity. Also, other factors such as publication and updates of population databases and thereby the interval for reclassification can influence the reclassification rates according to current ACMG criteria. To enable genetic variant classification and re-classification up to date, clinical geneticists play a critical role in the reinterpretation process with specific consideration of the cardiac phenotype, a key capability that genetic diagnostic laboratories primarily might not have.

**Clinical geneticists** as a part of an interdisciplinary cardiogenetic centre are responsible for providing, to the patient and family members, all the information regarding genetic testing and the implications of each possible result (e.g., patient surveillance and family risk assessment) as well as the possibility of reinterpretation if new evidence becomes available. Any variant re-classification should be communicated to the requesting healthcare professional and provided to variant carriers and/or affected family members to achieve a correct genetic diagnosis with its potential clinical consequences. As new cardiovascular genetics and genomics knowledge both continue to become integrated into healthcare, it will be essential to ensure that clinicians achieve particular key competencies and knowledge about the potential and limitations of genetics (and applied methods) in disease diagnostics, screening, prevention, prognostics, treatment, and counselling. Subsequently, the recent development of practice guidelines for clinicians and laboratories is essential for patient management and achievement of interdisciplinary clinical competencies in order to guide the incorporation of genetics and genomics into the practice of cardiovascular health care. Additionally, there are multiple training and certification programs available at different national and international levels, e.g. studentships, fellowships, workshops, online courses, case-based learning, and laboratory courses.

## 3. Cardiac arrhythmias with a genetic background – examples

### 3.1. Congenital Long-QT Syndrome (LQTS)

LQTS is characterized by a prolongation of the QT interval, typically measured in a baseline 12-lead or exercise ECG (recommended paper speed: 50 mm/s). These measured QT values have to be corrected for heart rate (by using Bazett’s formula to derive the corrected QTc value). A QTc of >450 ms (males) or >460 ms (females) is indicative for a LQTS but there is overlap with the normal population. The presence of intraventricular conduction delay such as complete right or left bundle branch block may limit the use of the QTc interval. The QTc interval can also be affected by many drugs. In contrast to congenital LQTS, drug-induced QTc prolongation (“acquired LQTS”) is often not genetic, and only in 10–15 % of cases can the drug be considered to have unmasked “hidden LQTS”. For many physicians, recognition of LQTS and accurate QT interval measurements are still difficult [18]. A weighted scoring system for the diagnosis of congenital LQTS, also called the ‘Schwartz score’, incorporates ECG criteria and other clinical and historical criteria; a diagnostic score (≥3.5 points) is diagnostic, but there is also an intermediate probability for disease (1.5 to 3 points); in some patients, the diagnosis of LQTS still may be uncertain after application of the diagnostic criteria outlined above. Consequently, in recent ESC guidelines an indicative genetic test was proposed to be diagnostic [19].

**Table 3:** Genes for LQTS (adopted from [10, 22])

**Isolated (cardiac) Forms** ClinGen MonDO: 0002442			
**LQTS**	**Core Genes** (Variant Detection Rate >1 %)	**Rare Genes (< 1 %)**	Mode of Inheritance
	*KCNQ1 (LQT1)*, *KCNH2 (LQT2)*, *SCN5A (LQT3)*	*CACNA1C, CALM1, CALM2, CALM3, KCNE1, KCNJ2, (RYR2)* *TRDN* (AR)	**AD, AR,** **sporadic**
**Syndromal Forms**			
	Gene	Additional Clinical Signs	Mode of Inheritance
Jervell and Lange-Nielsen syndrome (JLNS)	*KCNQ1,* *KCNE1*	*Congenital hearing loss*	**AR**
Timothy syndrome (TS)	*CACNA1C*	*Partial syndactylia, flat nasal bridge, low-set ears, small upper jaw, narrow upper lip, autism*	**AD,** **sporadic**
Andersen Tawil Syndrome (ATS)	*KCNJ2*	*Periodic paralysis, short stature, scoliosis, deep-set ears, hypertelorism, broad nasal root, micrognathia, clinodactyly, brachydactyly and syndactyly*	**AD**

AD: autosomal dominant; AR: autosomal recessive

Due to cascade family investigations and systematic genetic testing, it now has become clear that there are many LQTS variant carriers without symptoms. These asymptomatic but LQTS variant-positive patients still have congenital LQTS, even though the term “syndrome” might be misleading. In a series of 1,710 LQTS cases, the mean QTc interval was QTc 471 + 45 ms, but a large portion had a normal QTc interval below 460 ms (genetic subtypes: 47 % LQT1, 36 % LQT2 and 35 % LQT3) [20]. The overall risk for cardiac events or arrest might be lower and mainly was determined by the degree of QT interval prolongation and exposure to risk or event-triggering factors. A variant analysis is essential not only in patients with evident LQTS but also in asymptomatic family members with normal QTc interval (i.e., <440–460 ms) who have a tenfold increased risk of cardiac events in comparison to noncarriers.

The type of variant and the location of the variant within the particular channel domains have been proposed to impact on the underlying biophysical defect and, subsequently, on severity of the disease. However, the clinical utility of such information has not been validated yet. The obvious variability of clinical presentation among carriers of the same LQTS variant, even in the same family, initiated studies to investigate the role of i.e. single nucleotide polymorphisms (SNPs) for disease modulation. So far, rare SNPs alleles have been investigated in LQTS patients and in the general population and have been proposed to influence repolarization, but unlikely in drug-induced forms of QT interval prolongation. Further studies are needed to show the impact on clinical decision-making upon SNP genotyping, since the overall effect of minor SNPs alleles on the QTc interval usually is small (<5 ms).

In 2020, Adler *et al*. revalidated the disease causality and relationship of so far published genes for long-QT syndrome (LQTS) regarding the ClinGen requirements [21]. Only three genes (*KCNQ1*; chr. 11p15.5; sensitivity: ~35 % of all cases; *KCNH2,* chr. 7q36.1; sensitivity: ~30 %; and *SCN5A,* chr. 3p22.2; sensitivity: ~10–15 %) identified with definitive evidence. Additional genes associated with a syndromal forms of LQTS (+ extracardiac clinical signs) are: *CACNA1C (*definitive disease evidence for Timothy syndrome) with moderate evidence for typical LQTS. Limited disease genes for typical LQTS are *KCNJ2* (but definitive for syndromal Andersen-Tawil syndrome), *KCNE1* (strong for atypical LQTS) and *CAV3*. Another four genes associated with atypical LQTS, where prolongation is connected with neonatal heart block of autosomal-recessive inheritance had definitive or strong evidence (definitive: *CALM1* (calmodulin 1), *CALM2* (calmodulin 2)*, CALM3* (calmodulin 3); strong: *TRDN* (triadin))[21]. In case of *KCNE2*, this gene was finally disputed for LQTS.

**Table 4:** Genes for Brugada syndrome (adopted from [10, 22])

**Isolated Cardiac Forms** ClinGen MonDO: 0015263			
**Brugada Genes**	**Core Gene** (Variant Detection Rate >1 %)	**Rare Genes** (< 1 %)	Mode of Inheritance
	*SCN5A*		**AD**

### 3.2. Brugada Syndrome (BrS)

The Brugada syndrome is an autosomal dominantly inherited ion channel disorder with a variable phenotypic, the transient expression is often characterized bundle branch block and a characteristic downsloping ST-segment elevation in leads V1–V3. ECG changes might be hidden or transient, patients with typical ECG features who are asymptomatic and have no other clinical criteria (e.g., positive family history) have a so-called **Brugada ECG pattern**. When symptoms are present, i.e. typical ECG features together with experienced or survived sudden cardiac death, syncope or documented ventricular tachycardia patients are said to have the **Brugada syndrome (BrS)**. The typical ECG pattern needed for the diagnosis of Brugada syndrome (BrS) can occur provocable or incidentally (e.g. during class 1 antiarrhythmics) and/or triggered by fever or vagotonia; the so-called type 1 ECG, which is characterized by an ST-segment elevation (J-point elevation) of ≥2 mm in ≥1 lead accompanied by a descending ST segment and a symmetrical, negative T-wave. In the current ESC guidelines [19], Brugada syndrome is diagnosed in particular (expert class 1 recommendation) when a spontaneous type 1 ECG is present OR an inducible type 1 ECG (medication, fever, etc.) is present in a patient with sudden cardiac arrest or ventricular fibrillation. Affected patients often also have atrio-ventricular conduction disorder and corresponding ECG signs, as well as atrial and malignant ventricular arrhythmias. The prevalence of BrS is potentially overestimated and given as 1 in 2,000, with a higher prevalence in Asian countries. The symptomatic patients are usually men in the 3rd – 4th decade of life [23].

Due to the limited specificity of drug provocation tests in the diagnosis of BrS and several, potential phenocopies (e.g., mechanical RVOT compression, myocardial ischemia, electrolyte imbalances, hyperthermia, drug intoxications), a so-called ‘Shanghai Diagnostic Score’ has been proposed, where other clinical features are taken into account in addition to the type 1 ECG [24]. A score of ≥ 3.5 points corresponds to a definitive diagnosis of BrS, a score of 2–3 points to a possible or suspected diagnosis. In the context of risk stratification, the genetic finding (presence of a *SCN5A* gene variant), but also other polygenic gene markers (such as SNPs) may play a role in the future. In addition, genome-wide association studies in patients with BrS identified several genetic loci with common, non-coding but potentially regulatory variants (so-called polygenic risk scores, PRS) that were associated with the different phenotype manifestation of the disease, response to the ajmaline test, or the development of cardiac events. The clinical evaluation of these additional genetic tests is currently underway.

In 2019, a study by Campuzano *et al*. examined variants in 42 possible Brugada genes with regard to their potential disease pathogenicity. This study concluded that apart from *SCN5A* also *SLMAP*, *SEMA3A*, *SCNN1A,* and *SCN2B* should be included in the list of BrS related disease genes [25].

### 3.3. Catecholaminergic polymorphic ventricular tachycardia (CPVT) and Calcium Release Deficiency Syndrome (CRDS)

Catecholaminergic polymorphic ventricular tachycardia (CPVT) is a potentially malignant and heritable arrhythmia syndrome characterized by bidirectional or polymorphic VT during physical or emotional stress [26, 27]. It is estimated to affect 1 in 10,000 people with reported mortality rates as high as 30–50 % by the age of 35 years when left untreated, although most patients will present before the age of 10 [28]. The baseline ECG is usually normal, but during exercise (heart rate >120–140 bpm), typically monomorphic and later polymorphic or bidirectional premature ventricular beats (VES) occur. Some patients may have these during emotional rather than physical stress. Consequently, patients may experience syncope or cardiac arrest during a sustained polymorphic or bidirectional ventricular tachycardia or due to ventricular fibrillation (VF) that occurred secondarily. There still are differential diagnoses to be considered (e.g., arrhythmogenic cardiomyopathies (ACM), electrolyte disturbances, digitalis intoxication, VES at resting conditions). Risk factors for sudden death include documented VF, a family history of sudden death, and onset of symptoms in childhood. Beta receptor blockers are often given for primary prevention and should be used for secondary prevention, although the response is not uniform. Flecainide has been effectively added; in severe and highly symptomatic cases left cardiac sympathetic denervation (LCSD) is recommended. Patients with risk factors for sudden death and no response to pharmacological therapy often receive an ICD.

**Table 5:** Genes for Catecholaminergic Polymorphic Ventricular Tachycardia (CPVT) (adopted from [10, 22])

**Isolated (Cardiac) Forms** ClinGen MonDO: 0017990				
**CPVT Genes**		**Core Genes** (Variant Detection Rate >1 %)	**Rare Genes** (< 1 %)	Mode of Inheritance
	*RYR2,* *CASQ2*		*CALM1, CALM2,* *CALM3* *CASQ2,* *TRDN,* *TECRL* (AR)	**AD, AR**

The majority of CPVT cases (variant sensitivity, 50–60 %) were caused by heterozygous, non-synonymous pathogenic variants in the *RYR2* gene (ryanodine receptor 2, CPVT1)[29] that encodes the sarcoplasmic reticulum (SR) Ca^2+^ channel called ryanodine receptor because of its affinity for binding the alkaloid ryanodine. *RYR2* variants are inherited in an autosomal dominant manner whereas *CASQ2* pathogenic variants are rare (approximately 5 % of cases) and demonstrate autosomal recessive inheritance [30, 31]. Patients with *RYR2* pathogenic variants have similar clinical courses than those without; recessive forms of CPVT have a more severe clinical course and are more resistant to β-blocker therapy. In patients with no family history of CPVT, sporadic variants are likely to be the cause and may occur.

Using the ClinGen gene curation framework, definitive disease genes for CPVT are *RYR2*, *CASQ2, TECRL* and *TRDN* [32] where genes with a moderate evidence for disease causation in CPVT were the three calmodulin genes (*CALM1, CALM2, CALM3*), *TRDN* (triadin) and trans-2,3-enoyl-CoA reductase-like gene (*TECRL*). Other genes were disputed (*KCNJ2*, *PKP2* and *SCN5A*) or refused (ankyrin 2 [*ANK2*]) as causative for CPVT.

Most of the reported variants in *RYR2* are gain-of-function variants, but also a small part of loss-of-function variants are described. These loss-of-function variants are related to frameshifts, CNVs and secondary haploinsufficiency. In 2014, a deletion of exon 3 of *RYR2* was reported, associated with left ventricular non-compaction (LVNC); a second report a non-synonymous variant with familial cosegregation showed an unexpected clinical overlap between LVNC and atypical CPVT due to an in-vitro loss-of-function [33]. Furthermore, another *RYR2* gene variant reduced Ca2^+^ release and short-coupled ventricular torsade-de-pointes arrhythmias. During the last years, other pathogenic variants were characterized as *RYR2* loss-of-function and variant carriers did not show typical, clinical CPVT signs (in particular exercise-induced arrhythmias). To separate these different RYR2 in-vitro effects, the term Ca^2+^ Release Deficiency Syndrome (CRDS) has been recently established as a novel RYR2-related phenotypic spectrum [34].

### 3.4. Short-QT Syndrome (SQTS)

Short-QT syndrome (SQTS) is characterized by a very short-QT interval, absence of the ST segment, and symmetric, tall T-waves on the ECG (<330 ms, or <360 ms and symptoms). It is a very rare cardiac ion channel disease (< 1: 10,000) that can be associated with syncope, paroxysmal atrial fibrillation, ventricular fibrillation or sudden cardiac death. Diagnostic criteria are according to the

**HRS/EHRA/APHRS Consensus Recommendations** [35]: QTc ≤330 ms or QTc 330–360 ms + at least 1 additional diagnostic feature,
**ESC SCD Guidelines Consensus Recommendations** [36]: QTc ≤340 ms (Class 1 recommendation) or QTc 340–360 ms + at least 1 additional diagnostic feature,
**So-called Gollob criteria**, **SQTS score** [37]: ≥ 4 points (high probability), 2–3 points (intermediate probability),
**ESC VA/SCD Guidelines Consensus Recommendations** [19]: QTc ≤ 320 ms or QTc 320–360 ms + gene variant, family history, or survivor of sudden cardiac death (SCA) in ventricular tachycardia (VF, VT).

Clinical signs may be adrenergically mediated (increasing QTc shortening with higher heart rate)[38]; however, there are also SQTS patients where vagal-mediated paradoxical QTc interval shortening and symptoms of bradycardia occur. Implantation of an ICD with/without quinidine therapy is recommended for high-risk patients, regardless of genetic status. Life-threatening cardiac arrhythmias are equally common across different genotypes. Syncope are more frequent in male than in females whereas their symptoms (like atrial flutter, atrial fibrillation or palpitations) not. SQTS is highly lethal with sudden cardiac arrest/sudden cardiac death (SCA/SCD) mostly being the first clinical manifestation in about 30 % of SQTS patients. First choice of therapy is implantable cardioverter defibrillator (ICD), quinidine can be an effective pharmacological therapy. Exogenous causes or phenocopies for SQTS have still to be considered, e.g., congenital lactase deficiency, primary carnitine deficiency* (SLC22A5* gene), primary hyperparathyroidism or Klinefelter syndrome (47, XXY), acidosis, digitalis intoxication, hyperthermia, hyperkalemia, hypercalcemia, congenital adrenal hyperplasia, hyperthyroidism, vitamin A intoxication, milk-alkali syndrome, and treatment with rufinamide (anti-convulsive) or selective ATP potassium channel openers (pinacidil, levcromakalim).

**Table 6:** Genes for Short-QT Syndrome (SQTS) (adopted from [10, 22])

**Isolated (Cardiac) Forms** ClinGen MonDO: 0000453					
	**Core Genes** (Variant Detection Rate >1 %)		**Rare Genes** (< 1 %)		Mode of Inheritance
	*KCNH2, KCNQ1, SLC4A3*	*KCNJ2*			**AD**
**Syndromal Forms**					
Carnitine deficiency	*SLC22A5*			*Early childhood cardiomyo-pathy, muscle hypotension, failure to thrive, hypoglycemia, convulsion*	**AD**

SQTS is an autosomal dominant inherited disease. ClinGen expert panels currently consider only four genes as disease-causing genes [32]. In addition to the potassium channel genes *KCNH2* (SQT1), *KCNQ1* (SQT2) and *KCNJ2* (SQT3), the cardiac chloride-bicarbonate exchanger AE3 encoded by *SLC4A3* is a main cause for SQTS. While *KCNH2* and *KCNQ1* were classified as definite and moderate genes, respectively, *KCNJ2* and *SLC4A3* are only classified as moderate so far. With regard to the *SLC4A3* gene, two families have been identified with the identical variant. *SLC4A3*-related SQTS resulted from experimental models showing that the membrane anion exchange (bicarbonate vs. chloride) protein showed a reduced membrane localization of the mutant protein (harboring an amino acid exchange) leading to a loss-of-function by intracellular alcalinization and shortening of the cardiomyocyte action potential duration. Also, some syndromic cases have been noted with a *CACNA1C* loss-of-function variant.

An indicative molecular genetic finding (class 4 or 5 variant according to ACMG-) is considered an important diagnostic feature in the international recommendations (Gollob score: 2 points). In about 20 % of the index patients, such variants can be detected.

### 3.5. Other uncommon forms of inherited arrhythmias

Other familial forms of supra- and ventricular arrhythmia have been described. In contrast to the inherited arrhythmia syndromes (see 3.1. – 3.4.), there is no current disease-gene evaluation by the ClinGen Board, since for many of these uncommon inherited arrhythmias, there is currently little data on the variant detection rate, i.e. the pathogenic variant fraction, and on possible genotype-phenotype correlations.

The recommendation for genetic testing is not so strong, due to the often low rate of positive findings (e.g. in supraventricular arrhythmias). However, in individual disorders and with regard to a familial occurrence and clinical relevance by malignant forms of arrhythmias (e.g. IVF, unclear SCA, IVT), it can be relevant not only in an etiological context, but also relevant in family cascade screening by early detection of additional variant carriers. If there is a positive family history for one of the listed diseases, genotyping should be initiated.

## 4. Future directions of cardiogenetics

In cardiovascular medicine, genetic testing is an upcoming, but still underutilized tool in supporting of a cardiovascular diagnosis and risk management; this is applicable for some of the inherited arrhythmia syndromes and cardiomyopathies where genotype-phenotype are established. Currently, genetic testing is viewed as a highly specialized process reserved for cardiovascular genetic specialists. However, given the population prevalence and breadth of cardiovascular phenotypes for which genetic testing is warranted, the indications for genetic testing are more and more defined recently. However, a precise (i.e., non-genetic) diagnostic cardiovascular assessment is always essential before initiating a comprehensive and focussed genetic test with its subsequent consequences. In particular, disease phenocopies and borderline clinical presentations have to be considered. In consequence of an increasing awareness of the potential contribution of genetics to cardiovascular disorders, ongoing education of the cardiovascular community in these issues is an important key to increase clinical awareness of potential benefits and use of genetic testing. This should be accompanied by dedicated cardiogenetic education and cardiovascular medicine training programs. In order to improve patient specialized health care services and outcomes, the rapid knowledge growth in genetics and genomics has to be carefully considered, including re-evaluation of initial genetic findings in genes and their variants. This is, of course, challenging for health care professionals since it does not only integrate core competencies in genetics but also recent developments and published data, e.g. in-vitro data supporting gene causality or renewed disease relationship by re-evaluation of ClinGen [39] or technical advances using next-generation sequencing techniques. In recent years, also significant copy number or structural variations (CNV, SV), such as large deletions or duplications, have been recognized during genetic analysis as well as regulatory gene variants and are likely to be implemented as a part of a comprehensive and complex genetic analysis. On contrast, causal, deep intronic variants, balanced and unbalanced translocations, inversions, complex rearrangements, for example, are more rare and can primarily identified using whole genome sequencing (WGS) [3, 4] and additional chromosomal analysis. Cardiovascular genetics including the development of polygenic risk scores (PRS) will continue to pave the way for a further personalized medicine, taking inherited patient information and the individual clinical profile further into account.

**Table 7:** Other uncommon forms of inherited arrhythmias and genes (adopted from [10, 22])

**Idiopathic ventricular fibrillation (IVF)** **“Sudden cardiac arrest” (SCA), SCD, SIDS, SU(N)DS** **Idiopathic ventricular tachycardia (IVT)**
*Multi-gene panel investigation* *of cardiac ion channel and cardiomyopathy genes*
**Short-coupled Torsade de pointes-Tachycardia (scTDP)**
***RYR2, SCN5A***
**Multifocal ectopic Purkinje-related premature contractions and related cardiomyopathy (MEPPC)**
***SCN5A***
**Early repolarisation syndrome (ERS)**
*No validated or curated genes.*
**Atrial fibrillation (ATFB)** (idiopathic or familial)
***SCN5A, KCNQ1, MYL4, TTN***
**WPW Syndrome with HCM/LVH**
***PRKAG2,*** *mitochondrial DNA (Leigh syndrome)*
**Cardiac conduction disease (CCD/PCCD)**
***SCN5A, TRPM4,*** *GJA5, SCN1B*
**Cardiac conduction disease (CCD/PCCD)**, syndromal form
***LMNA****,* ***TNNI3K,*** *DES, DMD, DMPK, EMD, LAMP2, ZNF9, GLA, PRKAG2,* *NKX2-5, GJC1, TBX5, mitochondrial DNA*
**Sinus node disease (SND)**
***HCN4,*** ***KCNJ5,*** *GNB2, KCNQ1, RYR2, SCN5A*
**Sinus node disease (SND)**, syndromal form
***LMNA,*** *CACNA1D, EMD, GNB5, SGOL1*

CCD, “cardiac conduction disease”; PCCD, “progressive cardiac conduction disease”; SCA, “sudden cardiac arrest”; SCD, “sudden cardiac death”; SIDS, “sudden infant death syndrome”; SUDS, “sudden unexpected death syndrome”; SUNDS, “sudden unexpected nocturnal death syndrome”; HCM/LVH, hypertrophic cardiomyopathy, left ventricular hypertrophy.

## References

[1] Ryan T, Roberts JD (2024). Stem cell models of inherited arrhythmias. Nat Cardiovasc Res.

[2] Simons E, Loeys B, Alaerts M (2023). iPSC-Derived Cardiomyocytes in Inherited Cardiac Arrhythmias: Pathomechanistic Discovery and Drug Development. Biomedicines.

[3] Senthivel V, Jolly B, Vr A, Bajaj A, Bhoyar R, Imran M (2024). Whole genome sequencing of families diagnosed with cardiac channelopathies reveals structural variants missed by whole exome sequencing. J Hum Genet.

[4] Pagnamenta AT, Camps C, Giacopuzzi E, Taylor JM, Hashim M, Calpena E (2023). Structural and non-coding variants increase the diagnostic yield of clinical whole genome sequencing for rare diseases. Genome Med.

[5] Landstrom AP, Kim JJ, Gelb BD, Helm BM, Kannankeril PJ, Semsarian C (2021). Genetic Testing for Heritable Cardiovascular Diseases in Pediatric Patients: A Scientific Statement From the American Heart Association. Circ Genom Precis Med.

[6] Stiles MK, Wilde AAM, Abrams DJ, Ackerman MJ, Albert CM, Behr ER (2020). APHRS/HRS expert consensus statement on the investigation of decedents with sudden unexplained death and patients with sudden cardiac arrest, and of their families. Heart Rhythm.

[7] Schulze-Bahr E, Dettmeyer RB, Klingel K, Kauferstein S, Wolf C, Baba HA (2021). Postmortale molekulargenetische Untersuchungen (molekulare Autopsie) bei kardiovaskulären und bei ungeklärten Todesfällen. Der Kardiologe.

[8] Wilde AAM, Semsarian C, Marquez MF, Sepehri Shamloo A, Ackerman MJ, Ashley EA (2022). European Heart Rhythm Association (EHRA)/Heart Rhythm Society (HRS)/Asia Pacific Heart Rhythm Society (APHRS)/Latin American Heart Rhythm Society (LAHRS) Expert Consensus Statement on the state of genetic testing for cardiac diseases. Europace.

[9] (2022). 2022 ESC Guidelines for the management of patients with ventricular arrhythmias and the prevention of sudden cardiac death: Developed by the task force for the management of patients with ventricular arrhythmias and the prevention of sudden cardiac death of the European Society of Cardiology (ESC). European Heart Journal.

[10] Schulze-Bahr E, Klaassen S, Gerull B, von Kodolitsch Y, Landmesser U, Rieß O (2023). Gendiagnostik bei kardiovaskulären Erkrankungen. Die Kardiologie.

[11] Zeljkovic I, Gauthey A, Manninger M, Malaczynska-Rajpold K, Tfelt-Hansen J, Crotti L (2024). Genetic testing for inherited arrhythmia syndromes and cardiomyopathies: results of the European Heart Rhythm Association survey. Europace.

[12] Barc J, Tadros R, Glinge C, Chiang DY, Jouni M, Simonet F (2022). Genome-wide association analyses identify new Brugada syndrome risk loci and highlight a new mechanism of sodium channel regulation in disease susceptibility. Nat Genet.

[13] Walsh R, Lahrouchi N, Tadros R, Kyndt F, Glinge C, Postema PG (2021). Enhancing rare variant interpretation in inherited arrhythmias through quantitative analysis of consortium disease cohorts and population controls. Genet Med.

[14] Lahrouchi N, Tadros R, Crotti L, Mizusawa Y, Postema PG, Beekman L (2020). Transethnic Genome-Wide Association Study Provides Insights in the Genetic Architecture and Heritability of Long QT Syndrome. Circulation.

[15] Nauffal V, Morrill VN, Jurgens SJ, Choi SH, Hall AW, Weng LC (2022). Monogenic and Polygenic Contributions to QTc Prolongation in the Population. Circulation.

[16] Choi SH, Jurgens SJ, Haggerty CM, Hall AW, Halford JL, Morrill VN (2021). Rare Coding Variants Associated With Electrocardiographic Intervals Identify Monogenic Arrhythmia Susceptibility Genes: A Multi-Ancestry Analysis. Circ Genom Precis Med.

[17] Fernandez-Falgueras A, Coll M, Iglesias A, Tiron C, Campuzano O, Brugada R (2024). The importance of variant reinterpretation in inherited cardiovascular diseases: Establishing the optimal timeframe. PLoS One.

[18] Viskin S, Rosovski U, Sands AJ, Chen E, Kistler PM, Kalman JM (2005). Inaccurate electrocardiographic interpretation of long QT: the majority of physicians cannot recognize a long QT when they see one. Heart Rhythm.

[19] Zeppenfeld K, Tfelt-Hansen J, de Riva M, Winkel BG, Behr ER, Blom NA (2022). ESC Guidelines for the management of patients with ventricular arrhythmias and the prevention of sudden cardiac death. Eur Heart J.

[20] Mazzanti A, Maragna R, Vacanti G, Monteforte N, Bloise R, Marino M (2018). Interplay Between Genetic Substrate, QTc Duration, and Arrhythmia Risk in Patients With Long QT Syndrome. J Am Coll Cardiol.

[21] Adler A, Novelli V, Amin AS, Abiusi E, Care M, Nannenberg EA (2020). An International, Multicentered, Evidence-Based Reappraisal of Genes Reported to Cause Congenital Long QT Syndrome. Circulation.

[22] Schulze-Bahr E, Dittmann S (2024). Human Genetics of Cardiac Arrhythmias. Adv Exp Med Biol.

[23] Milman A, Andorin A, Gourraud JB, Postema PG, Sacher F, Mabo P (2018). Profile of patients with Brugada syndrome presenting with their first documented arrhythmic event: Data from the Survey on Arrhythmic Events in BRUgada Syndrome (SABRUS). Heart Rhythm.

[24] Antzelevitch C, Yan GX, Ackerman MJ, Borggrefe M, Corrado D, Guo J (2016). J-Wave syndromes expert consensus conference report: Emerging concepts and gaps in knowledge. J Arrhythm.

[25] Campuzano O, Sarquella-Brugada G, Fernandez-Falgueras A, Cesar S, Coll M, Mates J (2019). Genetic interpretation and clinical translation of minor genes related to Brugada syndrome. Hum Mutat.

[26] Lucet V, Grau F, Denjoy I, Do Ngoc D, Geiger K, Ghisla R (1994). [Long term course of catecholaminergic polymorphic ventricular tachycardia in children. Apropos of 20 cases with an 8 year-follow-up]. Arch Pediatr.

[27] Leenhardt A, Lucet V, Denjoy I, Grau F, Ngoc DD, Coumel P (1995). Catecholaminergic polymorphic ventricular tachycardia in children. A 7-year follow-up of 21 patients. Circulation.

[28] Abbas M, Miles C, Behr E (2022). Catecholaminergic Polymorphic Ventricular Tachycardia. Arrhythm Electrophysiol Rev.

[29] Priori SG, Napolitano C, Tiso N, Memmi M, Vignati G, Bloise R (2001). Mutations in the cardiac ryanodine receptor gene (hRyR2) underlie catecholaminergic polymorphic ventricular tachycardia. Circulation.

[30] Lahat H, Eldar M, Levy-Nissenbaum E, Bahan T, Friedman E, Khoury A (2001). Autosomal recessive catecholamine- or exercise-induced polymorphic ventricular tachycardia: clinical features and assignment of the disease gene to chromosome 1p13–21. Circulation.

[31] Bhuiyan ZA, Hamdan MA, Shamsi ET, Postma AV, Mannens MM, Wilde AA (2007). A novel early onset lethal form of catecholaminergic polymorphic ventricular tachycardia maps to chromosome 7p14-p22. J Cardiovasc Electrophysiol.

[32] Walsh R, Adler A, Amin AS, Abiusi E, Care M, Bikker H (2021). Evaluation of gene validity for CPVT and short QT syndrome in sudden arrhythmic death. Eur Heart J.

[33] Roston TM, Guo W, Krahn AD, Wang R, Van Petegem F, Sanatani S (2017). A novel RYR2 loss-of-function mutation (I4855M) is associated with left ventricular non-compaction and atypical catecholaminergic polymorphic ventricular tachycardia. J Electrocardiol.

[34] Steinberg C, Roston TM, van der Werf C, Sanatani S, Chen SRW, Wilde AAM (2023). RYR2-ryanodinopathies: from calcium overload to calcium deficiency. Europace.

[35] Priori SG, Wilde AA, Horie M, Cho Y, Behr ER, Berul C (2013). HRS/EHRA/APHRS expert consensus statement on the diagnosis and management of patients with inherited primary arrhythmia syndromes: document endorsed by HRS, EHRA, and APHRS in May 2013 and by ACCF, AHA, PACES, and AEPC in June 2013. Heart Rhythm.

[36] Harrell DT, Ashihara T, Ishikawa T, Tominaga I, Mazzanti A, Takahashi K (2015). Genotype-dependent differences in age ofmanifestation and arrhythmia complications in short QT syndrome. Int J Cardiol.

[37] Gollob MH, Redpath CJ, Roberts JD (2011). The short QT syndrome: proposed diagnostic criteria. J Am Coll Cardiol.

[38] Giustetto C, Scrocco C, Schimpf R, Maury P, Mazzanti A, Levetto M (2015). Usefulness of exercise test in the diagnosis of short QT syndrome. Europace.

[39] Tavtigian SV, Greenblatt MS, Harrison SM, Nussbaum RL, Prabhu SA, Boucher KM (2018). Modeling the ACMG/AMP variant classification guidelines as a Bayesian classification framework. Genet Med.

